# Chorea secondary to human immunodeficiency virus infection

**DOI:** 10.1055/s-0043-1771170

**Published:** 2023-10-04

**Authors:** André Eduardo de Almeida Franzoi, Amanda Maieski, Caio César Diniz Disserol, Helio Afonso Ghizoni Teive

**Affiliations:** 1 Universidade Federal do Paraná, Hospital de Clínicas, Departamento de Medicina Interna, Serviço de Neurologia, Curitiba PR, Brazil.; 2 Universidade Federal do Paraná, Hospital de Clínicas, Setor de Distúrbios do Movimento, Curitiba PR, Brazil.

A 55-year-old woman presented with facial and cervical chorea for 3 months (video). She had a previous history of traumatic right facial nerve palsy but no comorbidities or current medication use. Brain magnetic resonance imaging (MRI) was performed ( Figure 1 ). Cerebrospinal fluid analysis was unremarkable. Serum tests were positive for human immunodeficiency virus (HIV) type 1 with 1.877.056 viral copies and T-CD4 lymphocyte count of 35/mm ^3^ . Darunavir, ritonavir, dolutegravir, and lamivudine were initiated. After 4 months, chorea showed resolution ( Video 1 ). Chorea is a rare manifestation of HIV infection. 1 2 Differential diagnoses like neurosyphilis, Huntington disease, and Wilson disease should be ruled out. 2

**Video 1** Patient with chorea before treatment for HIV and without chorea after treatment with antiviral therapy.


**Figure 1:**
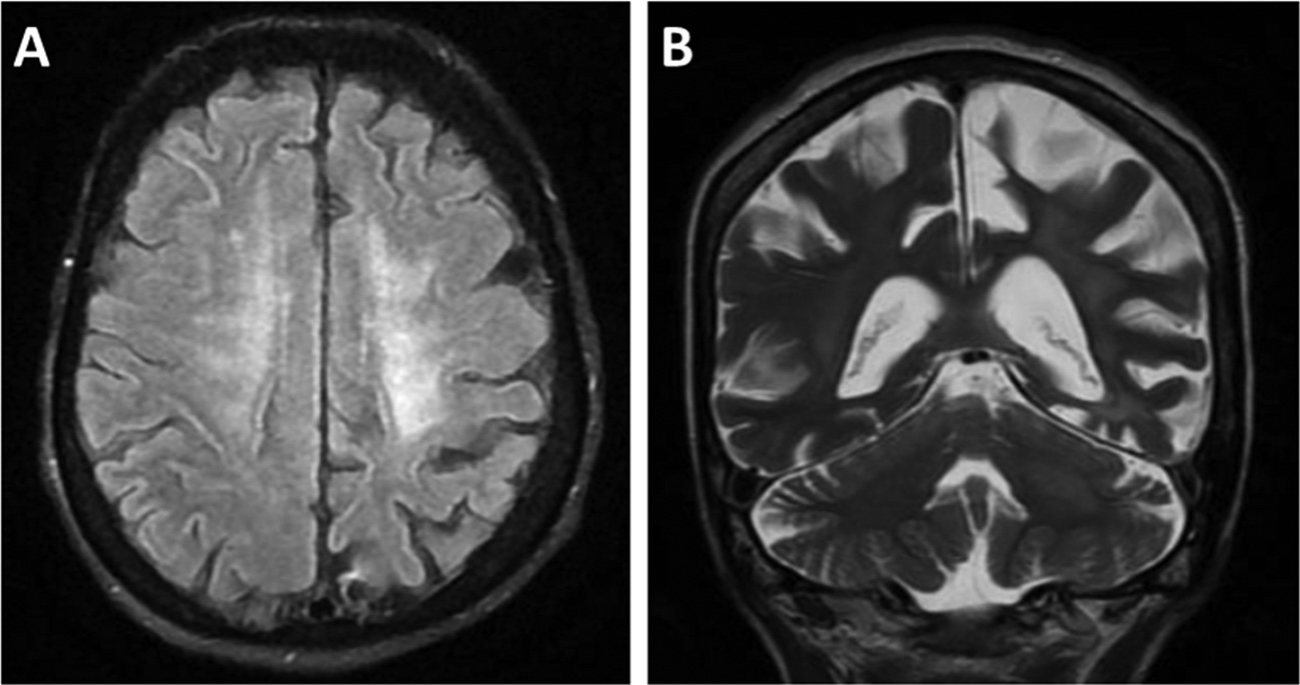
(A) Magnetic resonance imaging with axial T2/FLAIR sequence shows nonspecific hyperintensities in the white matter. (B) Coronal T2-weighted sequence shows brain volumetric reduction predominantly on the left parieto-occipital region, hyperintensities in the white matter, and moderate dilatation of supratentorial ventricular system with prominence of the cerebral sulci and basal cisterns.

## References

[1] Cardoso F HIV-related movement disorders: epidemiology, pathogenesis and management CNS Drugs 2002 16 10 663 668 10.2165/00023210-200216100-00002 12269860

[2] Rajan S Kaas B Moukheiber E Movement Disorders Emergencies Semin Neurol 2019 39 01 125 136 10.1055/s-0038-1677050 30743298

